# Temporal and spatial patterns of Leprosy in Uganda, 2020–2024: A nationwide surveillance analysis

**DOI:** 10.1371/journal.pntd.0014450

**Published:** 2026-07-02

**Authors:** Gertrude Abbo, Richard Migisha, Emmanuel Mfitundinda, Lilian Bulage, Geofrey Amanya, Alex Mulindwa, Rose Kengonzi, Stavia Turyahabwe, Henry Luzze, Benon Kwesiga, Alex Riolexus Ario

**Affiliations:** 1 Uganda Public Health Fellowship Program, Uganda National Institute of Public Health, Kampala, Uganda; 2 National Tuberculosis and Leprosy Program, Ministry of Health, Kampala, Uganda; University of Bremen: Universitat Bremen, GERMANY

## Abstract

**Introduction:**

Despite sustained elimination efforts, leprosy transmission persists in Uganda. The World Health Organization’s *Towards Zero Leprosy* strategy targets zero infections and Grade 2 Disability (G2D) by 2030. Uganda’s National TB and Leprosy Strategic Plan aims to reduce child cases to <3% and G2D to <5%. We assessed temporal trends and spatial distribution of leprosy in Uganda from 2020–2024 to evaluate progress toward these targets.

**Methods:**

We conducted a descriptive analysis using national surveillance data from the District Health Information System 2 (DHIS2). Newly diagnosed leprosy cases reported between 2020 and 2024 were analyzed by age, sex, clinical type, and disability grade. Incidence was calculated using annual mid-year population estimates from the Uganda Bureau of Statistics. Temporal trends were assessed using the Mann–Kendall test and Sen’s slope estimator. Spatial distribution of incidence, child cases, and G2D proportions was examined using QGIS at the regional and district levels.

**Results:**

Between 2020 and 2024, Uganda reported 1,935 new leprosy cases, with an overall incidence of 8.8 per 1,000,000 population. Multibacillary disease accounted for 86% of cases, and 21% presented with Grade 2 Disability (G2D). Children aged ≤15 years comprised 14% of cases, consistently exceeding the national elimination target of 3%. No significant monotonic trend was observed in overall incidence (p = 0.89), child proportion (p = 0.069), or G2D proportion (p = 0.14). Spatial analysis demonstrated persistent concentration of high incidence in the West Nile region, with 9 of 13 districts bearing the highest disease burden. Elevated G2D proportions were observed intermittently in the Teso and Ankole regions.

**Conclusion:**

Leprosy incidence in Uganda remained relatively stable from 2020 to 2024; however, persistently high proportions of multibacillary disease, child cases, and G2D indicate ongoing transmission and delayed diagnosis in specific regions. Targeted interventions, including strengthened contact tracing and active case detection in high-burden areas, are essential to accelerate progress toward national elimination goals.

## Introduction

Leprosy is a chronic infectious disease caused by *Mycobacterium leprae*, which primarily affects the skin, peripheral nerves, upper respiratory tract, and eyes. Delayed diagnosis and older age worsen the progression of physical deformities associated with the disease [1]. The disease has a lengthy and varied incubation period, typically averaging 5 years but potentially extending up to 20 years before symptoms appear [2]. Leprosy primarily spreads through direct or indirect contact between infected individuals and healthy people [3]. For treatment purposes, the World Health Organization (WHO) classifies patients into two categories: paucibacillary (PB), characterized by to five skin lesions, and multibacillary (MB), with six or more lesions. This classification guides appropriate treatment regimens and helps assess the risk of leprosy reactions and nerve damage [4].

Over 120 countries globally are leprosy endemic, with approximately 200,000 new cases reported annually [5]. The world officially achieved elimination of leprosy as a public health issue, defined as a prevalence of <1 per 10,000 population, in the year 2000, in line with the World Health Assembly Resolution 44.9, and in most countries by 2010 [6]. At the end of 2021, the global prevalence of leprosy was 16.9 per million population. By region, the highest prevalence was in Southeast Asia with 81,222 cases (39.4 per million), followed by the Americas with 25,053 cases (24.3 per million), Africa with 20,960 cases (18 per million), the Eastern Mediterranean with 4,206 cases (5.5 per million), and the Western Pacific with 2,360 cases (1.2 per million) [7].

Leprosy remains endemic in Uganda, with 40% of districts reporting at least one case to the National Tuberculosis and Leprosy Program (NTLP) as of 2016 [8]. In 2015/2016, 217 new cases were detected, 69% from the Northern region and 6% in children under 15, indicating ongoing transmission. By the financial year 2021/22, cases increased from 383 to 477, with the proportion of child cases slightly decreasing but still above the National Strategic Plan (NSP) targets [9]. The incidence of G2D also surged to 1.72 per million, over three times the NSP target [10].

Over the past 25 years, Uganda has made considerable progress in the fight against leprosy. The country achieved elimination of Leprosy as a public health problem in 1994 and has sustained this over the years. However, there has been a gradual increase of the number of cases registered in Uganda annually over the past five years, from 121 cases in 2017–388 cases reported in 2021. The number of new cases among children and those identified with major physical disabilities is high at 13% and 21.5%, respectively. The growing trend is due to the reinvigoration of leprosy control services and the ongoing transmission of leprosy in the community [11].

In 2021, the Ministry of Health launched the Community Awareness, Screening, Testing, and Treatment (CAST) Campaign to enhance case-finding for undiagnosed tuberculosis (TB) and leprosy cases, including those missed during the COVID-19 pandemic [12]. This initiative aimed to reduce community transmission and ultimately eliminate TB and leprosy in Uganda. In 2023, Uganda launched its Zero Leprosy Roadmap (2022–2030), a strategic framework designed to strengthen leprosy elimination efforts. Uganda’s National TB and Leprosy Strategic Plan 2020/21–2024/25 identified childhood leprosy and Grade 2 Disability (G2D) at diagnosis as key programme indicators. The plan aimed to reduce the proportion of newly diagnosed leprosy cases occurring among children from 8.6% in 2018 to <3% by 2024/25 and to reduce the proportion of patients presenting with G2D at diagnosis from 13.8% to <5% over the same period [10,13]. Persistently elevated childhood leprosy notifications may indicate sustained transmission, while a high proportion of G2D at diagnosis suggests delayed case detection. Assessing these indicators alongside temporal and spatial patterns of newly diagnosed cases is therefore important for identifying areas where intensified leprosy-control interventions are needed. We described the temporal and spatial patterns of newly diagnosed leprosy cases in Uganda from 2020 to 2024 and assessed progress against selected targets of the Uganda National TB and Leprosy Strategic Plan 2020/21–2024/25.

## Methods

### Ethics statement

Our study utilized routinely aggregated surveillance data with no personal identifiers in health facility quarterly reports, obtained from the DHIS2. The Uganda Public Health Fellowship Program is part of the National Rapid Response Team and has been granted permission to access and analyze surveillance data in the DHIS2 and other data, such as survey and field investigation data to inform decision-making in the control and prevention of outbreaks and public health programming. Additionally, the Ministry of Health has also granted the program permission to disseminate the information through scientific publications. We stored the abstracted dataset in a password-protected computer and only shared it with the investigation team. This activity was reviewed by the US CDC and was conducted consistent with applicable federal law and CDC policy. §§See, e.g., 45 C.F.R. part 46, 21 C.F.R. part 56; 42 U.S.C. § 241(d); 5 U.S.C. § 552a; 44 U.S.C. § 3501 et seq. All protocols were approved by the US CDC human subjects review board (The National Institute for Occupational Safety and Health Institutional Review Board) and the Uganda Ministry of Health, and were performed in accordance with the Declaration of Helsinki.

### Study area and setting

Our study was conducted using data from across Uganda, which is administratively divided into 15 regions: North Central, Tooro, South Central, Acholi, Lango, Karamoja, Kigezi, Teso, Bugisu, Bukedi, Busoga, Bunyoro, Kampala City, Ankole, and West Nile. These regions are further subdivided into 146 districts. Uganda has 155 hospitals, comprising 2 National Referral Hospitals,14 Regional Referral Hospitals (RRHs), and 139 General Hospitals (GHs). Leprosy diagnosis and treatment are provided free of charge in Uganda. Suspected leprosy cases are first seen at lower-level health facilities such as Health Centers II, III, and IV, before referral to hospitals [14]. Currently, six health facilities serve as dedicated leprosy treatment centers: five General Hospitals and one Health Center III, two are located in Eastern Uganda, three in Northern Uganda, and one is situated in Western Uganda [8].

### Case definitions and clinical classification

A “new leprosy case” was defined according to national guidelines as a patient newly diagnosed with leprosy during the reporting period who had not previously received multidrug therapy (MDT). Cases were identified through routine health facility reporting to the national District Health Information System (DHIS2).

Leprosy type was classified at the time of diagnosis using World Health Organization (WHO) operational criteria. Paucibacillary (PB) disease was defined as the presence of 1–5 skin lesions without detectable bacilli on slit-skin smear (where available), while multibacillary (MB) disease was defined as >5 skin lesions and/or positive slit-skin smear. In routine practice, classification was primarily based on clinical lesion count.

Disability grading was assessed at diagnosis using the WHO disability grading system. Grade 2 Disability (G2D) was defined as visible deformity or damage attributable to leprosy, including visible muscle weakness, clawing, foot drop, lagophthalmos, or severe visual impairment. Disability grading was performed by trained clinicians at the time of diagnosis and recorded in facility registers before initiation of treatment.

### Study design and data source

This study was designed as a descriptive ecological analysis of routinely collected national leprosy surveillance data reported in the DHIS2 from 2020 to 2024. The DHIS2 is a computer-based national health information system where health data are reported weekly, monthly, quarterly, and annually. Because DHIS2 relies on routine reporting, data completeness and accuracy may vary across districts. We assessed reporting consistency by reviewing the completeness of quarterly submissions and cross-checking annual totals.

Since 2012, leprosy cases have been reported quarterly to the Ministry of Health from 5,299 health facilities across 15 administrative health regions. Each facility uses a standardized reporting form to document leprosy cases by week, month, and quarter, categorizing them as new cases, G2D cases, MB and paucibacillary PB cases, and by age group (≥15 years and <15 years). These data are submitted to the Ministry of Health through the monthly Health Management Information System (HMIS) Form 105 and the quarterly HMIS Form 106 in the DHIS2.

### Study variables, data abstraction, and analysis

We collected data on leprosy cases categorized by age, sex, type of leprosy, and disability grade. Incidence was calculated by person (sex, age, and disability grade), place (districts), and time (cases reported by quarter). Leprosy incidence was defined as the number of new cases per 1,000,000 population per year. To determine the incidence of leprosy across different demographic groups and districts, we used the number of newly diagnosed cases as the numerator and the baseline population as the denominator. Annual mid-year population projections for 2020–2024 obtained from the Uganda Bureau of Statistics (UBOS) were used as denominators for incidence calculations [15]. Population estimates were disaggregated by sex (male/female), age group (<15 years and ≥15 years), district, and region. These denominators were used to calculate annual incidence rates per 1,000,000 population overall and by demographic subgroup. The denominator values used in the analysis are provided in S1 Table

We summarized national programme indicators annually to describe patterns over time and compare observed values with selected targets in Uganda’s National TB and Leprosy Strategic Plan 2020/21–2024/25. These annual indicators included the new-case detection rate, child-case proportion, child-case detection rate, proportion of newly diagnosed cases presenting with G2D, and G2D detection rate.

For temporal trend analysis, we used quarterly observations to provide sufficient time points and improve temporal resolution. The five-year study period yielded only five annual observations, which were insufficient for a robust monotonic trend assessment. Quarterly aggregation provided 20 observations from 2020 to 2024. We plotted quarterly new-case detection rates and programme indicators and assessed monotonic trends using the Mann–Kendall test. Sen’s slope estimator was used to quantify the magnitude and direction of change per quarter. Data analysis was conducted using R software. To assess recent programme performance, we compared the observed annual indicators with selected targets in Uganda’s National TB and Leprosy Strategic Plan 2020/21–2024/25. We used these comparisons to describe progress against national programme targets.

## Results

### Annual demographic and clinical characteristics of newly diagnosed leprosy cases, Uganda, 2020–2024

We identified a total of 1,935 leprosy cases from 2020 to 2024, of which 14% were children < 15 years of age, 55% were females, 86% were multi-bacillary, and 21% presented with Grade 2 Disability (G2D) (Table 1). The overall incidence was 8.8/1,000,000.

**Table 1:** Annual demographic and clinical characteristics of newly diagnosed leprosy cases, Uganda, 2020–2024.

Characteristic	2020 n (%)	2021 n (%)	2022 n (%)	2023 n (%)	2024 n (%)	Total n (%)
Total cases	238	468	591	344	294	1,935
**Sex**						
Male	126 (53)	217 (46)	263 (45)	144 (42)	124 (42)	874 (45)
Female	112 (47)	251 (54)	328 (55)	200 (58)	170 (58)	1,061 (55)
**Age group**						
<15 years	33 (14)	55 (12)	73 (12)	56 (16)	48 (16)	265 (14)
≥15 years	205 (86)	413 (88)	518 (88)	288 (84)	246 (84)	1,670 (86)
**Type of leprosy**						
Multibacillary	211 (89)	357 (76)	539 (91)	300 (87)	256 (87)	1,663 (86)
Paucibacillary	27 (11)	111 (24)	52 (9)	44 (13)	38 (13)	272 (14)
**Disability grade**						
G2D	92 (39)	86 (18)	101 (17)	83 (24)	52 (18)	414 (21)

Values are n (%); G2D: Grade 2 Disability.

### Progress against selected national leprosy programme targets

The annual new-case detection rate increased from 5.5 per 1,000,000 population in 2020 to a peak of 13.1 per 1,000,000 in 2022, before declining to 6.4 per 1,000,000 in 2024. The proportion of child cases remained above the national target of <3% throughout the study period, ranging from 12% to 16%. The child-case detection rate similarly peaked in 2022 at 3.5 per 1,000,000 children and declined to 2.3 per 1,000,000 in 2024. The proportion of newly diagnosed cases presenting with Grade 2 Disability (G2D) also remained above the national target of <5%, ranging from 17% to 39%. Although the G2D detection rate fluctuated, it declined overall from 2.1 per 1,000,000 population in 2020 to 1.1 per 1,000,000 in 2024. Multibacillary disease accounted for more than three-quarters of cases in every year, with no consistent temporal pattern (Table 2).

**Table 2:** Annual national leprosy programme indicators and progress against selected national targets, Uganda, 2020-2024.

Indicator	2020	2021	2022	2023	2024	National target by 2024/25
Newly diagnosed cases, n	238	468	591	344	294	–
New-case detection rate per 1,000,000 population	5.5	10.6	13.1	7.4	6.4	–
Child cases aged <15 years, n	33	55	73	56	48	–
Child-case proportion, %	14	12	12	16	16	<3
Child-case detection rate per 1,000,000 children	1.7	2.7	3.5	2.6	2.3	–
Cases presenting with G2D, n	92	86	101	83	52	–
Proportion presenting with G2D, %	39	18	17	24	18	<5
G2D detection rate per 1,000,000 population	2.1	2.0	2.2	1.8	1.1	–
Multibacillary cases, n	211	357	539	300	256	–
Multibacillary proportion, %	89	76	91	87	87	–

G2D: Grade 2 Disability. Rates were calculated using annual mid-year population estimates from the Uganda Bureau of Statistics. National programme targets are < 3% for child cases and <5% for newly diagnosed cases presenting with G2D by 2024/25.

### Regional variation in child-case and Grade 2 Disability proportions

The five-year average proportion of child cases exceeded the national target of <3% in several regions, particularly West Nile (18%), Karamoja (10%), Bukedi (8%), Busoga (7%), and Acholi (6%). The average proportion of newly diagnosed cases presenting with Grade 2 Disability (G2D) also exceeded the national target of <5% in most regions. The highest average G2D proportions were observed in Ankole (54%), Teso (41%), West Nile (26%), and Karamoja (20%) (S1 Fig).

### Quarterly trends in national new-case detection rates and Grade 2 Disability proportions

**Fig 1:**
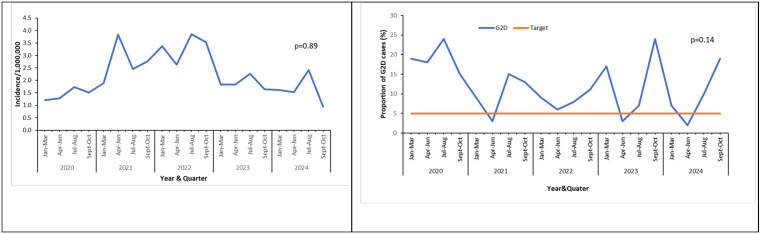
Temporal trends in the incidence of leprosy and proportion of G2D, Uganda, 2020–2024.

Quarterly leprosy incidence fluctuated between approximately 1.0 and 4.0 cases per 1,000,000 population during the study period (Fig 1a). Incidence increased through 2021 and peaked again in mid-2022 before declining in 2023–2024. However, no significant monotonic trend was detected (Mann–Kendall p = 0.89), indicating overall temporal stability in national incidence (S1 Fig). Similarly, the proportion of Grade 2 Disability (G2D) cases varied substantially across quarters, ranging from approximately 2% to 24% (S1 Fig). Although a weak downward tendency was observed (p = 0.14), the trend was not statistically significant. G2D proportions frequently exceeded the 5% programmatic target, with marked short-term variability rather than a sustained decline (S1 Fig).

Quarterly leprosy incidence fluctuated between approximately 1.0 and 4.0 cases per 1,000,000 population during the study period (Fig 1a). Incidence increased through 2021 and peaked again in mid-2022 before declining in 2023–2024. However, no significant monotonic trend was detected (Mann–Kendall p = 0.89), indicating overall temporal stability in national incidence (Fig 1a).

Similarly, the proportion of Grade 2 Disability (G2D) cases varied substantially across quarters, ranging from approximately 2% to 24% (Fig 1b). Although a weak downward tendency was observed (p = 0.14), the trend was not statistically significant. G2D proportions frequently exceeded the 5% programmatic target, with marked short-term variability rather than a sustained decline (Fig 1b).

**Fig 2:**
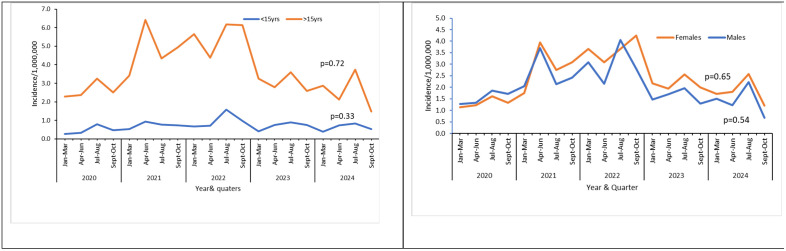
Temporal trends of leprosy incidence by age and sex, Uganda, 2020–2024.

Overall incidence among children aged <15 years was 0.7 per 1,000,000 population, compared with 3.7 per 1,000,000 among individuals aged ≥15 years (Fig 2). Incidence among those aged ≥15 years peaked in 2021–2022 at approximately 6–6.5 per 1,000,000, then declined in 2023–2024. In contrast, incidence among children remained substantially lower, fluctuating below 1.6 per 1,000,000 (Fig 2). Despite these fluctuations, no statistically significant monotonic trend was observed for either age group (Mann–Kendall p = 0.72 for ≥15 years; p = 0.33 for <15 years), indicating overall temporal stability in age-specific incidence patterns (Fig 2).

Overall, the incidence of leprosy was higher among females (2.4 per 1,000,000) compared to males (2.0 per 1,000,000) (Fig 2). Incidence in both sexes increased during 2021–2022, peaking at approximately 3.5–4.0 per 1,000,000, before declining in 2023–2024. Although females recorded slightly higher incidence than males in several quarters, the differences were small and inconsistent over time. Mann–Kendall analysis demonstrated no statistically significant monotonic trend for females (τ = 0.08, p = 0.65) or males (τ = −0.11, p = 0.54). Theil–Sen estimates further indicated minimal annual change, confirming overall temporal stability in sex-disaggregated incidence (Fig 2).

**Fig 3:**
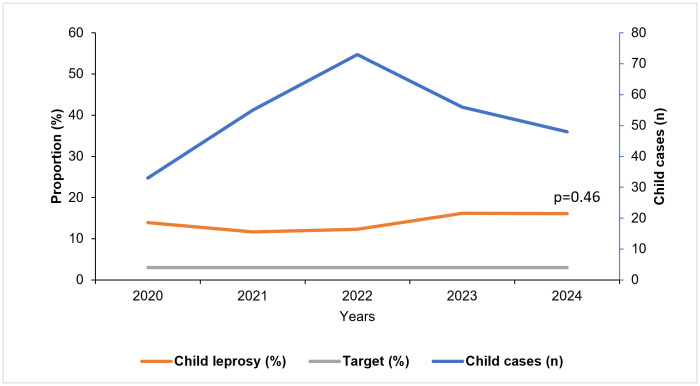
Temporal trend in the proportion of child leprosy cases (<15 years), Uganda, 2020–2024.

The quarterly proportion of child leprosy cases fluctuated between 9% and 22% over the study period and remained consistently above the national target of 3% at all time points (Fig 3). Peaks were observed in mid-2020 (~19%), late-2022 (~18%), late-2023 (~20%), and mid-2024 (~22%), while the lowest values occurred in early-2022 (~10%). Despite short-term variability, the overall pattern showed a weak upward tendency, although the Mann–Kendall test indicated no statistically significant monotonic trend (τ = 0.30, p = 0.069) (Fig 3).

**Fig 4:**
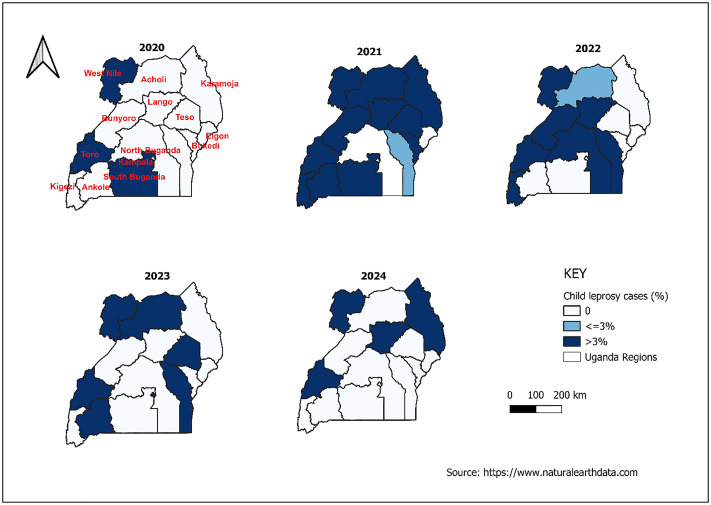
Spatial distribution of the proportion of child leprosy cases (<15 years), Uganda, 2020–2024.

Regions exceeding the national child leprosy target of 3% were geographically clustered in Northern and Eastern Uganda throughout the study period (Fig 4) West Nile exceeded 3% in all five years, indicating persistent clustering. Busoga, Bukedi, and parts of Northern Uganda exceeded 3% in at least three consecutive years, while most Central and Southwestern regions remained ≤3% or reported no child cases. Overall, the number of regions above the 3% threshold ranged from 4 to 7 regions annually, demonstrating sustained spatial concentration of child cases in specific geographic areas (Fig 4).

Choropleth maps show the proportion of child cases among newly diagnosed leprosy cases by region. Regions are categorized according to the national elimination target (≤3%) and above-target (>3%). Persistent exceedance of the threshold suggests ongoing transmission.

**Fig 5:**
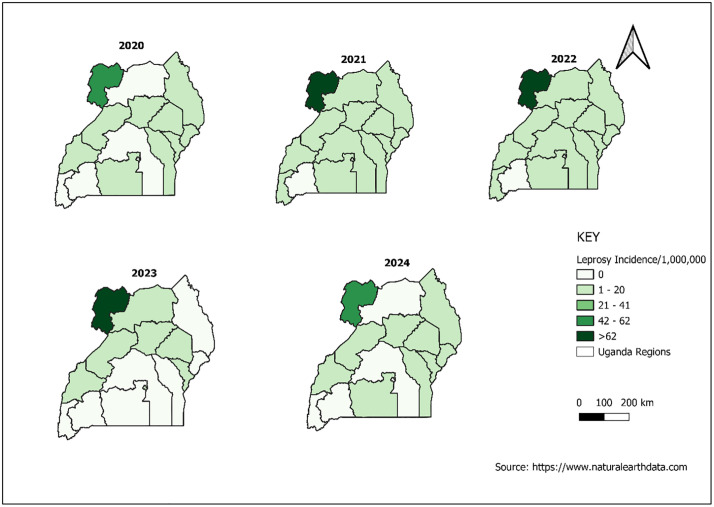
Spatial distribution of newly diagnosed leprosy cases, Uganda, 2020–2024. Source: https://www.naturalearthdata.com.

Incidence was concentrated in Northern Uganda throughout the study period. West Nile remained in the highest incidence category (>62 cases per 1,000,000) over the five years (Fig 5). Karamoja and Busoga recorded elevated incidence (≥42 per 1,000,000) for ≥3 years, whereas Central and Southwestern regions consistently reported low incidence (≤20 per 1,000,000). Each year, 1–3 regions fell within the highest incidence category, demonstrating sustained geographic clustering (Fig 5).

**Fig 6:**
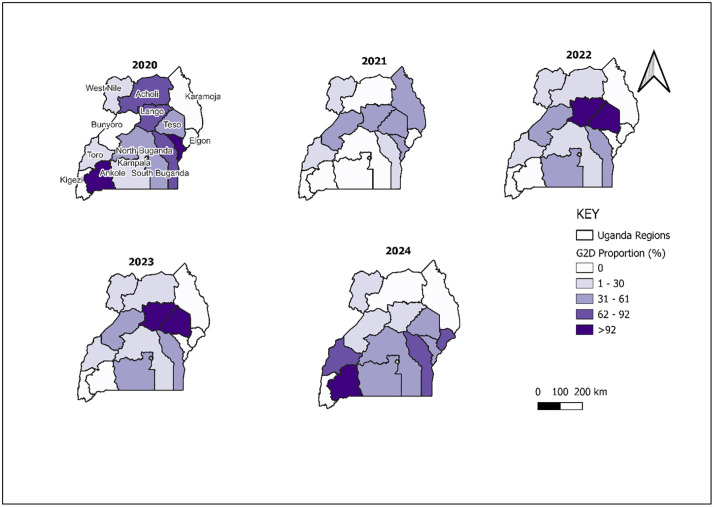
Proportion of leprosy cases diagnosed with Grade 2 Disability, Uganda, 2020–2024. Source: https://www.naturalearthdata.com.

Across the study period, G2D proportions demonstrated marked geographic concentration in specific regions rather than uniform distribution nationwide. High G2D proportions (>62%) occurred intermittently and were largely confined to Teso and Ankole, with only one region occupying the highest category (>92%) in any given year. Most regions consistently remained within the lower categories (≤61%), and no sustained nationwide decline in G2D distribution was observed (Fig 6).

## Discussion

From 2020 to 2024, Uganda reported nearly 2,000 leprosy cases, with an overall incidence of 9 per 1,000,000 population. Most cases were multibacillary, female, and occurred among individuals aged 15 years and above. Notably, 14% of cases were in children under 15 years, consistently exceeding the national elimination target of less than 3%. G2D remained high, averaging 21%, suggesting delays in diagnosis and treatment. The spatial analysis showed persistently high incidence in the West Nile region, with emerging hotspots in the Southwestern and Northeastern regions. Several districts, particularly in central, northeastern, and southwestern Uganda, reported very high G2D rates, further underscoring gaps in early case detection and access to care.

Progress toward Uganda’s national leprosy programme targets remained limited. Child cases accounted for 12%–16% of annual notifications, exceeding the national target of <3%, while 17%–39% of newly diagnosed cases presented with G2D, above the < 5% target. Although the G2D detection rate declined from 2.1 to 1.1 per 1,000,000 population between 2020 and 2024, these persistent gaps suggest ongoing transmission and delayed case detection. These findings underscore the need to strengthen active case detection, contact screening, and timely management, particularly in regions with persistently elevated indicators.

The overall incidence of leprosy in Uganda over the five years was relatively low, with an approximately 9 cases per 1,000,000 population reported. The observed decline in incidence after 2022, while encouraging, was not statistically significant and could reflect underreporting or temporary gains. Continued surveillance, combined with community-based interventions like the CAST campaign [16] and the implementation of the Zero Leprosy Roadmap ([17]), are critical to reversing these trends. The CAST campaign was aimed at identifying undiagnosed cases, including those missed during the COVID-19 pandemic [16], which could explain the rise in reported cases during this period. Despite the heightened case detection efforts, the incidence of leprosy remained way below the threshold for public health elimination, defined as a prevalence of <1 per 10,000 population by the World Health Organization [18].

Of particular concern is the increasing proportion of child leprosy cases, which averaged 13% over the study period and rose to 17% by 2024, substantially exceeding the WHO 2030 target of a 90% reduction in child case rates per million population [18]. Notably, this upward trend in child cases does not necessarily mirror the overall trend in leprosy incidence. The observed increase may partly reflect misdiagnosis or delayed recognition by health workers [19]. Nevertheless, rising child leprosy cases strongly suggest ongoing and recent transmission of *Mycobacterium leprae* within communities, particularly among vulnerable pediatric populations [20] The occurrence of leprosy in children is a recognized marker of recent transmission and highlights gaps in health system capacity to interrupt exposure pathways [21]. These findings underscore the need to strengthen contact screening, community health education, and the delivery of post-exposure prophylaxis to household and school contacts in high-burden settings, as well as routine health worker orientation on the signs and symptoms of leprosy, to reduce the risk of misdiagnosis and delayed detection.

The observed upward trend in G2D prevalence in Uganda from 2020 to 2024, with a notable surge in 2022, reflects ongoing challenges in early leprosy detection and timely treatment. The increase in districts reporting G2D prevalence >90%, particularly in the Eastern and Central regions, suggests delays in diagnosis and continued community transmission. Similar trends have been documented in other endemic countries. In India, a significant proportion of new leprosy cases presented with G2D, indicating missed opportunities for early detection and intervention [22]. The sharp rise in G2D prevalence in 2022 may also be partially explained by the indirect effects of the COVID-19 pandemic, which disrupted routine health services and reduced case detection globally [23]. Additionally, a multi-country review found a decline in new leprosy case notifications during the pandemic, followed by a rebound that included an increase in the proportion of G2D cases, likely due to delayed diagnosis [24].

The proportion of MB leprosy cases, which are more likely to transmit the disease increased during the study period. The increasing proportion of MB cases may reflect a higher level of active transmission in certain regions, further emphasizing the need for intensified case detection and treatment efforts [25]. These findings align with previous reports indicating that the majority of leprosy cases in Uganda are MB, which is more infectious and poses a greater challenge to control efforts [26].

Spatially, leprosy incidence and G2D clustering in Central, Eastern, and Northeastern Uganda over the years may reflect regional disparities in health system performance, access to leprosy services, or social determinants of health [27]. These findings reinforce the need for regionally tailored responses, including active case-finding, contact tracing, and decentralized rehabilitation services [28].

Our findings reveal critical public health and policy gaps in leprosy control in Uganda, particularly the persistently high burden of multibacillary cases, child leprosy, and G2D, all of which point to delayed diagnosis and ongoing community transmission. The consistent occurrence of new leprosy cases, child cases, and G2D above national targets, highlights an urgent need to strengthen active case finding, contact tracing, and early detection strategies, especially in high-burden regions like West Nile, Busoga, Bukedi, and Karamoja. National policies should prioritize decentralizing leprosy services, integrating leprosy surveillance into routine health systems, and enhancing community awareness and health worker training to improve timely diagnosis, and interrupt transmission.

### Study limitations

While our study provides valuable insights into the trends and distribution of leprosy in Uganda, there are some limitations to consider such as the use of surveillance data which may have been incomplete or inconsistently reported across districts, particularly in remote or underserved areas with limited diagnostic capacity. This could have led to an underestimation of the true leprosy burden. To mitigate this, we analyzed multi-year national data from 2020 to 2024 to capture broader trends and applied spatial analysis to identify consistent hotspots and emerging regions of concern. Secondly, reliance on aggregated routine surveillance data limited the ability to assess individual-level risk factors or explore social determinants of delayed diagnosis. To address this, we focused on internationally recognized indicators such as the proportion of child cases, multibacillary forms, and G2D as proxies for transmission intensity and program performance. Additionally, because incidence estimates were based on routinely notified newly diagnosed cases, incomplete case detection or reporting may have resulted in underestimation of the true incidence, particularly in remote or underserved areas with limited diagnostic capacity. Finally, the five-year surveillance period was insufficient for formal application of the WHO Leprosy Elimination Monitoring Tool and classification of regions across elimination phases. Longer-term analyses using the WHO LEMT framework and more detailed regional data are needed to assess progress towards interruption of transmission and elimination of disease.

## Conclusion

Uganda continued to report new leprosy cases from 2020 to 2024. The West Nile region consistently recorded the highest new-case detection rates, while elevated G2D proportions occurred intermittently in selected regions, particularly Ankole and Teso. The proportions of child cases and newly diagnosed cases presenting with G2D remained above the national programme targets, indicating continued transmission and delays in diagnosis. Strengthening active case detection, contact screening, timely referral, and post-exposure prophylaxis in high-burden areas will be important for accelerating progress towards national elimination goals. Moreover, further research is critical to identify the factors contributing to persistent incidence, particularly in high-burden regions, to inform more effective and locally tailored strategies.

## Supporting information

S1 Table Annual denominator populations used for incidence calculations, Uganda, 2020–2024. (PDF)

S1 Fig Regional leprosy incidence and key elimination indicators by region, Uganda, 2020–2024. (TIFF)

S1 Data Dataset used for the analysis of temporal and spatial patterns of leprosy in Uganda, 2020–2024. (RAR)

## Abbreviations

CDC: Centers for Disease Control and Prevention
MOH: Ministry of Health
RRH: Regional Referral Hospital
NTLP: National Tuberculosis and Leprosy Program
PB: Paucibacillary
MB: Multibacillary
G2D: Grade 2 Disability
DHIS2: District Health Information System version 2
NSP: National Strategic Plan
CAST: Community Awareness, Screening, Testing, and Treatment to end TB and Leprosy
