# Supplementary material for: Temporal and spatial patterns of Leprosy in Uganda, 2020–2024: A nationwide surveillance analysis
Source: PLoS Negl Trop Dis. 2026 Jul 2;20(7):e0014450. doi: 10.1371/journal.pntd.0014450 (PMC13345463; doi:10.1371/journal.pntd.0014450)
Supplement: S1 Table — (PDF) [file pntd.0014450.s001.pdf]

Supporting Information

S1_ Table. Annual denominator populations used for incidence calculations, Uganda, 2020–2024.

Year	Total Population	Male	Female	<15 years*	≥15 years*
2020	42,885,900	21,025,091	21,860,809	19,727,514	23,158,386
2021	44,009,227	21,575,518	22,433,709	20,244,244	23,764,983
2022	45,162,640	22,140,694	23,021,946	20,774,814	24,387,826
2023	46,346,114	22,720,204	23,625,910	21,319,213	25,026,901
2024	45,905,417†	22,379,433	23,525,984	21,116,492	24,788,925

Population denominators were obtained from the Uganda Bureau of Statistics (UBOS) annual mid-year population projections for 2020–2023 and the National Population and Housing Census 2024 Final Report. Denominators were stratified by sex and age group (<15 years and ≥15 years) for subgroup-specific incidence calculations
