# Supplementary figures and images for: Temporal and spatial patterns of Leprosy in Uganda, 2020–2024: A nationwide surveillance analysis
Source: PLoS Negl Trop Dis. 2026 Jul 2;20(7):e0014450. doi: 10.1371/journal.pntd.0014450 (PMC13345463; doi:10.1371/journal.pntd.0014450)

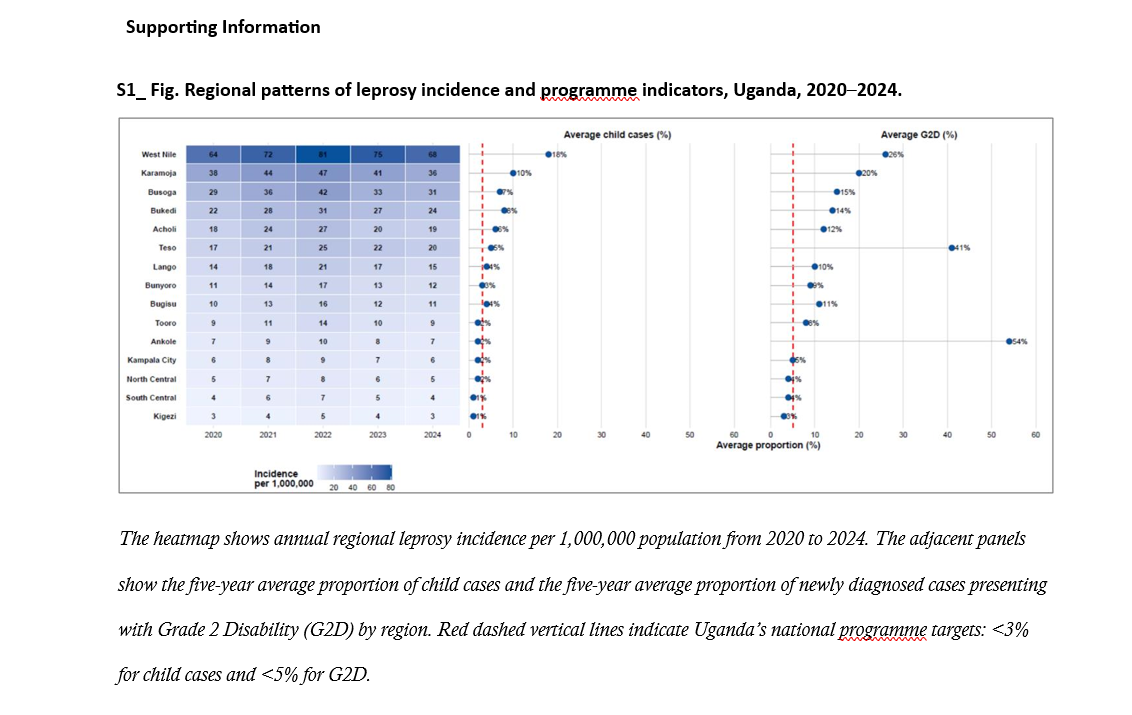

Supplement: S1 Fig — (TIFF) [file pntd.0014450.s002.tiff]
