# Supplementary material for: Temporal and spatial patterns of Leprosy in Uganda, 2020–2024: A nationwide surveillance analysis
Source: PLoS Negl Trop Dis. 2026 Jul 2;20(7):e0014450. doi: 10.1371/journal.pntd.0014450 (PMC13345463; doi:10.1371/journal.pntd.0014450)
Supplement: Attachment — Submitted filename: Reviewer Report for Manuscript PNTD.docx [file pntd.0014450.s004.docx]

**Reviewer Report for Manuscript PNTD-D-26-00058:**

**"Temporal and spatial patterns of Leprosy in Uganda, 2020-2024: A nationwide surveillance analysis"**

**Overall Assessment**

This manuscript presents a timely and policy-relevant analysis of leprosy surveillance data in Uganda from 2020 to 2024. The topic aligns well with the scope of PLOS Neglected Tropical Diseases, and the study addresses important questions regarding progress toward national and global elimination targets. The analysis is largely descriptive but provides useful insights into trends, age/sex distributions, disability grades, and geographic patterns. However, the manuscript in its current form requires major revisions before it can be considered suitable for publication. Several methodological, structural, and presentation issues must be addressed to strengthen clarity, rigor, and interpretability.

## Major Concern

1. **Title and Journal Alignment**

The title currently mentions "PLOS ONE Neglected Tropical Diseases" (line 7). This is incorrect; the target journal is **PLOS Neglected Tropical Diseases**. The title should be revised to reflect the journal's naming convention.

2. **Abstract Structure and Clarity**

- The abstract contains several incomplete sentences and formatting issues (e.g., lines 22–26).

- The term "prevalence" is used inconsistently; in line 26, "prevalence (\\~92%)" appears to refer to a regional proportion of cases, not prevalence as epidemiologically defined. This must be clarified.

- The conclusion in the abstract is somewhat vague and could be more specific to the study’s findings.

3. **Methods Section**

**- Data source and reporting**: While DHIS2 is described, there is no discussion of data quality, validation procedures, or potential reporting biases (e.g., underreporting in remote areas). This is critical for interpreting the observed trends.

- **Statistical analysis:** The Mann-Kendall test is appropriately chosen for trend analysis, but the results are presented without confidence intervals or measures of variability. Although the authors have reported p-values from their analysis, complementary analysis like Theil-Sen Estimator (Sen's Slope) would be handy in illustrating the trend magnitude (effect size).

- **Incidence calculation**: The use of 2024 population estimates as denominators for all years may introduce inaccuracies. The incidence should be calculated using the estimated mid-year population for each year.

- **Spatial analysis**: The description of GIS methods is minimal. Clustering methods (e.g., Moran’s I) should be applied to statistically identify hotspots rather than relying solely on visual inspection.

A sub-analysis for the West Nile region would be helpful. It would answer the question: Is the incidence leprosy uniformly distributed in the West Nile region? This would enable targeted interventions within the region.

Spatial analysis of the child leprosy cases would add a lot value to this paper, since it would point to where leprosy transmission is on-going.

4. **Results Presentation**

- **Figures and tables:**

- Table 1 is incomplete; it only shows counts and percentages for sex, type, and G2D but lacks annual breakdowns, or regional data. Annual breakdowns are needed for temporal trends while regional data is needed for spatial trends.

- The results text often repeats the same statistics in multiple sections, reducing readability.

- **Spatial findings**: The description of shifting hotspots is qualitative. Quantitative measures of spatial autocorrelation should be provided.

The spatial distribution of G2D (based on proportion of G2D among New Leprosy cases) is potentially misleading given that some regions report too few cases.

5. **Discussion Limitations**

- The discussion appropriately contextualizes findings but could better integrate the spatial and temporal results. For example, the emergence of new high-incidence areas (e.g., Toro in 2023) is noted but not sufficiently interpreted in terms of health system or community factors. Sub-analysis of weather the trend observed in these regions was for the entire district or a few districts would be very informative.

- Trends in overall incidence (line 120-125) show no pattern while trends of the proportion of child leprosy cases (line 161-168) show a statistically significant trend. Presence of child cases points towards on-going transmission that is not limited the child population. The authors must add a detailed discussion section reconciling the flat overall incidence trend with the rising proportion of child cases. This discussion must include explicit consideration of diagnostic challenges and the possibility of surveillance artefacts.

- The impact of the CAST campaign and COVID-19 disruptions is mentioned, but a more critical appraisal of how these may have influenced detection rates (e.g., increased screening vs. diagnostic delays) is needed.

- The mapping of G2D proportions should be interpreted with caution in regions with few annual cases, as estimates are unstable. The apparent high rates in some districts (e.g., >90%) may reflect small denominators. Nevertheless, the persistent spatial overlap between regions with high incidence and those with elevated G2D proportions suggests that diagnostic delays are widespread in high-transmission zones. The authors must:

1. Acknowledge this limitation prominently.

2. Re-analyze the spatial G2D data using appropriate statistical methods (smoothing) and/or presentation formats (showing absolute numbers alongside proportions).

3. Tone down definitive claims about geographic patterns in low-case regions.

6. **Ethical and Data Availability Statements**

- The data availability statement indicates that data are available upon request and with permission from UPHFP. This aligns with PLOS policy, but authors should confirm that data will be made available without restriction upon acceptance, as required.

- The ethical statement references US CDC and Uganda MOH approvals, which is appropriate

## Minor Concerns

**Writing and formatting:**

- Several typographical errors and inconsistent spacing exist (e.g., lines 9–10, 25–26).

- Abbreviations should be defined at first use (e.g., CAST in line 65 is defined later in the abbreviations list).

- The references are incomplete in the submitted PDF (many URLs are truncated or non-functional).

- **Repetitive content**: The background and introduction cover similar ground; consider condensing.

- **Targets and indicators**: The national targets for child cases (<3%) and G2D (<5%) are clearly stated, but their derivation or alignment with WHO thresholds could be briefly explained.

**Recommendation: Major Revision**

**Justification:**

The manuscript addresses an important public health issue with national and global relevance, and the analysis is based on a substantial national dataset. However, the current version has significant methodological gaps, incomplete results presentation (missing figures), and structural issues that hinder its scientific rigor and clarity. With careful revision—particularly in the methods, results presentation, and discussion—the study could make a valuable contribution to the leprosy elimination literature. I recommend **major revision** with the expectation that the authors will:

1. Clarify and expand the methods section, especially regarding data quality, statistical approaches, and spatial analysis.

2. Provide a complete Table 1 with annual and demographic breakdowns.

3. Strengthen the discussion by better integrating spatial and temporal findings and addressing limitations more explicitly.

4. Ensure consistent and accurate use of epidemiological terms (incidence vs. prevalence).

5. Correct formatting, referencing, and language issues throughout.
