# Supplementary material for: Temporal and spatial patterns of Leprosy in Uganda, 2020–2024: A nationwide surveillance analysis
Source: PLoS Negl Trop Dis. 2026 Jul 2;20(7):e0014450. doi: 10.1371/journal.pntd.0014450 (PMC13345463; doi:10.1371/journal.pntd.0014450)
Supplement: Attachment — Submitted filename: Point by Point Response to Reviewers revised 19.3.26.docx [file pntd.0014450.s006.docx]

**Point-by-Point Response to Reviewers**

**March 19^th^ ,2026**

Anil Fastenau, M.D., M.Sc.
Guest Editor
PLOS Neglected Tropical Diseases

**Manuscript ID:** PNTD-D-26-00058

**Re: Temporal and spatial patterns of leprosy in Uganda, 2020–2024: A nationwide surveillance analysis**

Dear Anil Fastenau,

We sincerely thank you for the thorough and constructive review of our manuscript. Your comments have substantially improved the clarity, rigor, and interpretability of our study. We have carefully revised the manuscript and addressed all comments point-by-point below. All changes are highlighted in the revised manuscript. For any further clarifications and information, please don’t hesitate to contact us.

Sincerely,

Gertrude Abbo (Corresponding Author)

**Reviewer Comments and Author Responses**

**Major Concerns**

**1. Title and Journal Alignment**

Comment:

The title currently mentions "PLOS ONE Neglected Tropical Diseases." This is incorrect.

**Response:**

Thank you for identifying this error. We corrected the journal name throughout the manuscript.

**Action taken:**

Replaced with “PLOS Neglected Tropical Diseases” (Title page). Line 7

**2. Abstract Structure and Clarity**

Comment:

Abstract contains incomplete sentences and inconsistent use of “prevalence.” The conclusion is vague.

**Response:**

We fully revised the abstract for clarity, completeness, and precision. We replaced incorrect uses of “prevalence” with “proportion” where appropriate and clarified epidemiological terminology. We also strengthened the conclusion to clearly reflect key findings and actionable implications.

**Action taken:**

Abstract rewritten to improve structure, terminology, and specificity (Abstract section).Line 9-37

**Reviewer #1 comments**

We sincerely thank the reviewer for the thorough and constructive feedback, which has helped strengthen the methodological rigor, clarity, and interpretability of the manuscript. Each comment is addressed in detail below.

1. **Use of DHIS2 data and data quality considerations**

The authors used DHIS2 for their analysis but there is no discussion on data quality and reporting biases that are critical for interpreting the observed trends.

**Response:**
We agree that discussion of data quality is essential when using routine surveillance data. We have now expanded the Methods section to describe DHIS2 reporting and potential sources of bias, including underreporting in remote districts, variability in diagnostic capacity, delayed case notification, and the influence of intensified case-finding activities. processes (Lines 134-137)

The Discussion section has also been strengthened to acknowledge how these factors may affect observed temporal and spatial patterns, particularly in low-incidence settings. (Line 329)

2. **Statistical analysis and lack of effect size measures**

**Reviewer comment:**
The Mann–Kendall test is appropriately chosen for trend analysis, but the results are presented without confidence intervals or measures of variability. Complementary analysis, such as the Theil–Sen estimator, would be useful.

**Response:**
We agree with this observation. In addition to reporting Mann–Kendall p-values, we have now incorporated Sen’s slope (Theil–Sen estimator) to quantify the magnitude and direction of trends. Trend magnitude estimates are now reported for key indicators, including overall incidence, the proportion of children with leprosy, and the proportion with Grade 2 Disability, improving interpretability beyond statistical significance alone.(Lines 182-221, 246-251)

3. **Incidence calculation using a single population denominator**

**Reviewer comment:**
The use of 2024 population estimates as denominators for all years may introduce inaccuracies. Incidence should be calculated using mid-year population estimates for each year.

**Response:**
We agree and thank the reviewer for highlighting this issue. Annual incidence rates have been recalculated using year-specific mid-year population estimates for 2020–2024 obtained from the Uganda Bureau of Statistics. All incidence-related results, tables, and figures have been updated accordingly. This has been clarified in the methods section as well (Lines 150-152)

4. **Spatial analysis and reliance on visual inspection**

**Reviewer comment:**
The description of GIS methods is minimal, and clustering methods (e.g., Moran’s I) should be applied rather than relying solely on visual inspection.

**Response:**
We appreciate this comment and have expanded the description of spatial methods to improve clarity and transparency. The study was designed as a descriptive surveillance analysis aimed at programmatic targeting rather than inferential spatial modelling. To strengthen the analysis, we introduced quantitative hotspot classification by defining persistent hotspots as regions remaining in the highest incidence category for three or more consecutive years. (Line 156-162)This approach provides an objective, reproducible measure of spatial clustering suitable for routine surveillance data. We also explicitly acknowledge in the Discussion that formal spatial autocorrelation statistics were not applied and note this as a limitation. (Line 379-381)

5. **Need for a West Nile sub-analysis**

**Reviewer comment:**
A sub-analysis for the West Nile region would help determine whether leprosy incidence is uniformly distributed.

**Response:**
We agree and have conducted a district-level sub-analysis for the West Nile region. This analysis demonstrated that incidence was not uniformly distributed across districts; several districts consistently recorded high incidence over multiple years. The results are now presented in a dedicated subsection with district-level maps and quantitative descriptions, providing actionable insight for targeted interventions within the region. (Line 290-299)

6. **Spatial analysis of child leprosy cases**

**Reviewer comment:**
Spatial analysis of child leprosy cases would add value by identifying areas of ongoing transmission.

**Response:**
We agree with the reviewer and have added a spatial analysis of child leprosy cases (<15 years), a recognized indicator of recent transmission. Annual regional proportions were mapped using the national elimination threshold (>3%), and persistence of above-threshold regions across consecutive years was quantified. The findings highlight sustained clustering of child cases in specific northern and eastern regions, and these results are now clearly described in the Results and integrated into the Discussion. (Lines 260-269)

**Response to Reviewer #2**

We thank the reviewer for the thoughtful and constructive feedback. The comments have helped improve the clarity, methodological transparency, and internal consistency of the manuscript. Each point is addressed below.

**1. Objective does not conclude a hypothesis; clarify a priori expectations or position as hypothesis-generating**

**Reviewer comment:**
The objective does not conclude a hypothesis. Consider explicitly stating a priori expectations or positioning this as hypothesis-generating.

**Response:**
We appreciate this observation. The study was designed as a national ecological surveillance analysis to describe temporal and spatial patterns rather than to test a predefined causal hypothesis. To clarify this positioning, we have revised the Introduction to explicitly state that the analysis is hypothesis-generating and intended to inform programmatic decision-making. This clarifies the analytical intent and aligns the inferential methods with the descriptive surveillance framework. (Line 130-132)

**2. Clarify definitions of “new leprosy cases,” PB/MB classification, and disability grading**

**Reviewer comment:**
Please elaborate on the underlying definitions for “new leprosy cases,” and describe how PB/MB and disability grading were measured and classified, and whether this was assigned at diagnosis.

**Response:**
We have expanded the Methods section to define case classifications clearly. A “new leprosy case” is now explicitly defined as an individual newly diagnosed during the reporting period who had not previously received multidrug therapy. PB and MB classifications were based on World Health Organization operational criteria, primarily determined at diagnosis using clinical lesion count (≤5 lesions for PB; >5 lesions for MB and/or positive smear where available). Disability grading was assessed at diagnosis using the WHO disability grading system, with Grade 2 Disability defined as visible deformity or damage attributable to leprosy. These definitions and the timing of classification at diagnosis are now clearly described. (Lines 117-128)

**3. Justification for trend-testing approach and clarification of quarterly vs annual data**

**Reviewer comment:**
Provide justification for the trend-testing approach. It is not clear whether quarterly data were used, and annual data (n=5) may be underpowered.

**Response:**
We agree that clarification was necessary. The manuscript has been revised to clearly distinguish between analyses conducted using quarterly time points (n=20) and those using annual summaries (n=5). Trend analyses were performed using quarterly data to improve statistical power and better capture short-term variability. The Methods section now explicitly states this, and figure captions have been revised to reflect the time unit used in each analysis. We also justify the use of the non-parametric Mann–Kendall test, which is appropriate for monotonic trend detection in surveillance time series without assuming normality. Where annual analyses were presented, we acknowledge the limited power and interpret findings alongside effect size estimates (Sen’s slope). Lines 184-186, 188-190,208 and Line 244)

**4. Use of single-year population denominator**

**Reviewer comment:**
Using 2024 census estimates as denominators for all years may bias rates.

**Response:**
We agree and have corrected this issue. Incidence rates were recalculated using year-specific mid-year population estimates for 2020–2024. This ensures accurate temporal comparisons and avoids bias associated with a single denominator across multiple years. All incidence-related tables and figures have been updated accordingly and clarified further in the methods section (Lines 151-153)

**5. GIS mapping details and terminology**

**Reviewer comment:**
Provide more detail on GIS methods (shapefile source, mapping unit, classification method). Avoid “hotspot” language unless tied to formal methods.

**Response:**
We have expanded the GIS methods section to include:

- The source of shapefiles (national administrative boundary shapefiles obtained from the Uganda Bureau of Statistics).
- The mapping unit (region and district levels). (Lines 157-158)

We have also revised the terminology throughout the manuscript to avoid the use of “hotspot” or “cluster” unless supported by explicit operational definitions. Where spatial persistence was described, the manuscript now uses neutral language such as “regions remaining in the highest incidence category for ≥3 consecutive years,” rather than implying statistical clustering. Lines 288-290, 294-295, 306-309)

**Response to Reviewer #3**

We thank the reviewer for the positive assessment of the study objective and design, and for the comment regarding techniques and indicators.

**Comment:**

The study design is sound, but the presentation of the data and the conclusions use obsolete techniques and indicators.

**Response:**
We appreciate this observation and have carefully reviewed the analytical approaches and indicators used in the manuscript. The study was designed as a national ecological surveillance analysis using routinely collected data, and therefore employed methods that are standard and widely recommended for trend detection in public health surveillance systems.

Specifically:

- The Mann–Kendall test was used because it is a non-parametric method appropriate for detecting monotonic trends in surveillance time-series data without assuming normality.
- The analysis has now been strengthened by incorporating Sen’s slope (Theil–Sen estimator) to quantify trend magnitude, thereby complementing p-values with interpretable effect sizes.
- Incidence calculations have been recalculated using year-specific mid-year population denominators.
- Definitions of key indicators (new case, PB/MB classification, Grade 2 Disability) have been clarified and aligned with current WHO operational standards.

The indicators used, including incidence, child proportion (<15 years), and Grade 2 Disability, remain central metrics in the World Health Organization’s leprosy monitoring framework and national elimination targets. These indicators were selected to ensure alignment with international surveillance standards and programmatic relevance.

We have revised the manuscript to clarify the rationale for methodological choices and to ensure that conclusions are appropriately framed within the limitations of surveillance data.
