# Supplementary material for: Temporal and spatial patterns of Leprosy in Uganda, 2020–2024: A nationwide surveillance analysis
Source: PLoS Negl Trop Dis. 2026 Jul 2;20(7):e0014450. doi: 10.1371/journal.pntd.0014450 (PMC13345463; doi:10.1371/journal.pntd.0014450)
Supplement: Attachment — Submitted filename: Point by Point Response to Reviewers V4.docx [file pntd.0014450.s007.docx]

**Point-by-Point Response to Reviewers**

**May 28^th^ , 2026**

Anil Fastenau, M.D., M.Sc.
Guest Editor
PLOS Neglected Tropical Diseases

**Manuscript ID:** PNTD-D-26-00058

**Re: Temporal and spatial patterns of leprosy in Uganda, 2020–2024: A nationwide surveillance analysis**

Dear Dr. Fastenau,

We sincerely thank the reviewers for the thorough and constructive review of our manuscript. We have carefully revised the manuscript and addressed all comments point-by-point below. All changes are highlighted in the revised manuscript. For any further clarifications and information, please don’t hesitate to contact us.

Sincerely,

Gertrude Abbo (Corresponding Author)

Reviewer #1

Methods

Comment 1: Study design and assessment of progress towards elimination

The authors state in line 77–78 the objective of the study as to evaluate Uganda’s progress towards leprosy elimination. Use of the WHO Leprosy Elimination Monitoring Tool (LEMT) would be more appropriate if they had data for a longer duration. The LEMT would visualize the temporal and spatial trends and clearly show the progress of the regions towards elimination. For instance, which regions have progressed from phase 1 (until interruption of transmission) to phase 2 (interruption of transmission until elimination of disease). If the authors opt not to use the LEMT, they should at the minimum compare the rates per million with the elimination targets for 2023 or 2025.

**Response:**
We thank the reviewer for this important recommendation. We agree that the WHO Leprosy Elimination Monitoring Tool provides a comprehensive framework for assessing longer-term progress towards interruption of transmission and elimination of disease, including classification of regions across elimination phases. However, our analysis covered five years of routinely collected surveillance data from 2020 to 2024. We considered this period insufficient for formal LEMT-based classification of regions across elimination phases.

Because the primary purpose of this study was to assess recent national programme performance, we revised the manuscript to benchmark the observed indicators against selected targets in Uganda’s National TB and Leprosy Strategic Plan 2020/21–2024/25. This national framework is directly aligned with the study period. We also added annual incidence rates per million population for the principal programme indicators.

We added the following text to the final paragraph of the Background:

“Uganda’s National TB and Leprosy Strategic Plan 2020/21–2024/25 identified childhood leprosy and Grade 2 Disability (G2D) at diagnosis as key programme indicators. The plan aimed to reduce the proportion of newly diagnosed leprosy cases occurring among children from 8.6% in 2018 to <3% by 2024/25 and to reduce the proportion of patients presenting with G2D at diagnosis from 13.8% to <5% over the same period.” Lines 67-71

We also revised the study objective as follows:

“We described the temporal and spatial patterns of newly diagnosed leprosy cases in Uganda from 2020 to 2024 and assessed progress against selected targets of the Uganda National TB and Leprosy Strategic Plan 2020/21–2024/25.”

**Location:** Background, lines 84-86

To strengthen the assessment of programme performance, we added Table 2, which presents annual national indicators, including the overall incidence rate, child-case incidence rate, G2D incidence rate, absolute case numbers, and proportions. The table also presents the selected national targets of <3% for child cases and <5% for newly diagnosed cases presenting with G2D.

**Location:** Results, Table 2, lines 189-190.

We further acknowledged the limitation of the five-year surveillance period:

“Finally, the five-year surveillance period was insufficient for formal application of the WHO Leprosy Elimination Monitoring Tool and classification of regions across elimination phases. Longer-term analyses using the WHO LEMT framework and more detailed regional data are needed to assess progress towards interruption of transmission and elimination of disease.”

**Location:** Study limitations, lines 353–357.

Comment 2: Population denominators and reproducibility

In line 121–125, the authors report using annual mid-year population estimates to calculate the incidence rates. They however do not provide a reference [full citation] or actual denominator values, making it hard to reproduce the results. The appropriate reference should include the mid-year population estimates together with the disaggregation by sex (male/female), age (<15 and ≥15), and the population of the regions.

**Response:**
We thank the reviewer for highlighting this important reproducibility issue. We revised the Methods to clarify that annual mid-year population projections from the Uganda Bureau of Statistics were used as denominators for the incidence calculations. We also clarified that population estimates were disaggregated by sex, age group, district, and region.

The revised text reads:

“Annual mid-year population projections for 2020–2024 obtained from the Uganda Bureau of Statistics (UBOS) were used as denominators for incidence calculations. Population estimates were disaggregated by sex (male/female), age group (<15 years and ≥15 years), district, and region. These denominators were used to calculate annual incidence rates per 1,000,000 population overall and by demographic subgroup.”

**Location:** Methods, lines 136–141.

We also added Supplementary Table and figure S1, which presents the annual total-population, sex-specific, and age-specific denominator values used in the analysis as well as the .

**Location:** Supplementary Table and figure S1, lines 173–174.

Results

Comment 3: Transparency of the geographical analysis

Table 1 is incomplete [lacks geographical data], making the spatial analysis presented not transparent.

**Response:**
We thank the reviewer for this observation. Table 1 was retained as a descriptive summary of the annual demographic and clinical characteristics of newly diagnosed leprosy cases. To improve transparency of the geographical analysis, we added Supplementary Figure 1, which presents annual regional incidence rates and five-year average regional child-case and G2D proportions. The incidence values are displayed directly within the regional heatmap cells.

The figure legend states:

“The heatmap shows annual regional leprosy incidence per 1,000,000 population from 2020 to 2024. The adjacent panels show the five-year average proportion of child cases and the five-year average proportion of newly diagnosed cases presenting with Grade 2 Disability (G2D) by region. Red dashed vertical lines indicate Uganda’s national programme targets: <3% for child cases and <5% for G2D.”

**Location:** Supplementary Figure 1, lines 587-592

We also retained the annual choropleth maps showing the spatial distribution of child-case proportions, regional incidence rates, and G2D proportions.

**Location:** Figures 4–6, lines 266–299.

Comment 4: Child-case proportions should not be presented without absolute numbers or incidence rates

Temporal trends on the proportion of child leprosy cases have been presented instead of absolute numbers or incidence of child leprosy cases. The proportion of child leprosy cases can increase even if child cases are stable or declining, if adult cases decline faster [or decrease even if child cases are rising, if adult cases rise faster].

**Response:**
We agree that proportions alone may not adequately reflect temporal changes in childhood leprosy burden because they are influenced by fluctuations in adult notifications. We therefore added annual absolute numbers and incidence rates for childhood leprosy alongside the child-case proportions.

The revised Results state:

“The proportion of child cases remained above the national target of <3% throughout the study period, ranging from 12% to 16%. The child-case detection rate similarly peaked in 2022 at 3.5 per 1,000,000 children and declined to 2.3 per 1,000,000 in 2024.”

**Location:** Results, lines 164–168.

Table 2 now reports annual child-case numbers, proportions, and incidence rates per 1,000,000 children.

**Location:** Results, Table 2, lines 178–186.

Comment 5: Regional G2D proportions may be misleading where notification counts are small

Spatial distribution of the proportion of leprosy cases diagnosed with Grade 2 Disability has been presented. However, given the variation in the denominator across the regions, the analysis may be misleading. That is, regions with too few notifications may appear to have a disproportionately higher burden of disability due to leprosy.

**Response:**
We thank the reviewer for this important observation. We agree that regional G2D proportions may appear disproportionately high where the number of notified cases is small. We therefore added the following cautionary statement:

“Regional G2D proportions should be interpreted cautiously because regions with few notified cases may appear to have disproportionately high percentages.”

**Location:** Results, lines 216-217.

Comment 6: Annual case numbers versus quarterly trend figures

The authors present number of annual cases in Table 1. However, in Figures 1–3, they presented quarterly incidence rates. Figures 1–3 can thus not be reproduced using data in Table 1. Unless the authors are interested in seasonal trends, use of annual cases would increase clarity for readers.

**Response:**
We thank the reviewer for this helpful comment. We agree that the original manuscript did not adequately explain the complementary purposes of the annual and quarterly analyses.

We retained quarterly observations for the temporal trend analyses because the study covered five years, yielding only five annual observations. Quarterly aggregation provided 20 observations and improved temporal resolution for the Mann–Kendall test and Sen’s slope estimator. The quarterly analyses were used to assess monotonic trends rather than seasonal patterns.

To improve transparency and reproducibility, we added Table 2, which presents annual national programme indicators, including annual incidence rates, child-case numbers and rates, G2D numbers and rates, multibacillary proportions, and the selected national targets.

The revised Methods state:

“We summarized national programme indicators annually to describe patterns over time and compare observed values with selected targets in Uganda’s National TB and Leprosy Strategic Plan 2020/21–2024/25.” Lines 145-148

and:

“For temporal trend analysis, we used quarterly observations to provide sufficient time points and improve temporal resolution. The five-year study period yielded only five annual observations, which were insufficient for a robust monotonic trend assessment. Quarterly aggregation provided 20 observations from 2020 to 2024. We plotted quarterly new-case detection rates and programme indicators and assessed monotonic trends using the Mann–Kendall test. Sen’s slope estimator was used to quantify the magnitude and direction of change per quarter.”

**Location:** Methods, lines 149–154.

Discussion and conclusion

Comment 7: Discussion should show progress against elimination targets

The authors stated in line 77–78 that the objective of the study is to evaluate the progress towards leprosy elimination. However, the discussion does not clearly show what progress has or has not been made since it is not anchored to elimination targets or sub-targets. The WHO’s “Towards Zero Leprosy” strategy (2021–2030) explicitly targets a 90% reduction in the child-case rate per million population by 2030, not a reduction in the proportion of child cases. The authors attempt in lines 241–243 to compare proportions of child leprosy with the 2030 target [in rates per million population], which is confusing. The discussion would be enriched if the authors commented on whether the data [annual incidence of cases, rate of new cases with G2D per million population, and new child cases per million population] compare with the sub-targets of 2023/2025.

**Response:**
We appreciate this clarification. We revised the manuscript to distinguish between proportions and incidence rates and to anchor the interpretation primarily to Uganda’s National TB and Leprosy Strategic Plan 2020/21–2024/25. Table 2 now presents annual incidence rates per million population, child-case incidence rates per million children, G2D incidence rates per million population, and the corresponding proportions.

Location: Results lines 189-191

We added the following paragraph to the Discussion:

“Progress toward Uganda’s national leprosy programme targets remained limited. Child cases accounted for 12%–16% of annual notifications, exceeding the national target of <3%, while 17%–39% of newly diagnosed cases presented with G2D, above the <5% target. Although the G2D detection rate declined from 2.1 to 1.1 per 1,000,000 population between 2020 and 2024, these persistent gaps suggest ongoing transmission and delayed case detection. These findings underscore the need to strengthen active case detection, contact screening, and timely management, particularly in regions with persistently elevated indicators.”

**Location:** Discussion, lines 341–346.

Comment 8: Conclusion should state the temporal and spatial patterns and progress towards elimination

In line 291–297, the authors need to clearly indicate if there are any temporal or spatial trends or patterns and what progress has been made towards leprosy elimination, if any.

**Response:**
We agree and revised the Conclusion to state the principal temporal and spatial patterns and the progress made towards selected national programme targets.

The revised Conclusion reads:

“Uganda continued to report new leprosy cases from 2020 to 2024. The West Nile region consistently recorded the highest new-case detection rates, while elevated G2D proportions occurred intermittently in selected regions, particularly Ankole and Teso. The proportions of child cases and newly diagnosed cases presenting with G2D remained above the national programme targets, indicating continued transmission and delays in diagnosis. Strengthening active case detection, contact screening, timely referral, and post-exposure prophylaxis in high-burden areas will be important for accelerating progress towards national elimination goals. Moreover, further research is critical to identify the factors contributing to persistent incidence, particularly in high-burden regions, to inform more effective and locally tailored strategies.”

**Location:** Conclusion, lines 412–420.

Reviewer #2

Comment 1: Figure cross-reference confusion

Lines 185–190 describe the spatial distribution of child leprosy cases but repeatedly cite “Figure 5” when this content corresponds to Figure 4. This appears to be a residual numbering error from the revision and should be corrected before publication.

**Response:**
We thank the reviewer for identifying this residual numbering error. We corrected the child-case spatial-map references to Figure 4 and renumbered the subsequent spatial figures sequentially.

The revised text now states:

“Regions exceeding the national child leprosy target of 3% were geographically clustered in Northern and Eastern Uganda throughout the study period (Figure 4).”

**Location:** Results, lines 230–238.

The subsequent regional incidence and G2D spatial maps are now presented as Figures 5 and 6, respectively.

**Location:** Results, lines 284–289.

Comment 2: Mild overclaim in the Conclusion

Line 291–292 states “an increasing rate of child case detection” as a confirmed finding, but the Mann–Kendall result for the child proportion trend was p = 0.069 — not statistically significant. The language should be softened to “a non-significant upward tendency” to stay consistent with the Results section.

**Response:**
We agree and removed the statement that child-case detection increased as a confirmed finding. The revised Conclusion now states that the child-case proportion remained above the national programme target without claiming a statistically significant increasing trend.

The revised text reads:

“The proportions of child cases and newly diagnosed cases presenting with G2D remained above the national programme targets, indicating continued transmission and delays in diagnosis.”

**Location:** Conclusion, lines 416–419.

Reviewer #3

Comment

The analysis is sound.

**Response:**
We thank the reviewer for this encouraging assessment.

We again thank the editor and reviewers for their thoughtful comments, which substantially improved the clarity, reproducibility, and programmatic relevance of the manuscript.
