# Supplementary material for: Temporal and spatial patterns of Leprosy in Uganda, 2020–2024: A nationwide surveillance analysis
Source: PLoS Negl Trop Dis. 2026 Jul 2;20(7):e0014450. doi: 10.1371/journal.pntd.0014450 (PMC13345463; doi:10.1371/journal.pntd.0014450)
Supplement: Peer review history

# Peer review: Decision Letter 0

**DOI:** 10.1371/journal.pntd.0014450.r001

**Authors:** Stuart Blacksell, Anil Fastenau

1 Feb 2026

PNTD-D-26-00058

Temporal  and spatial patterns of Leprosy in Uganda, 2020–2024: A nationwide surveillance analysis

PLOS Neglected Tropical Diseases

Dear Dr. Abbo,

Thank you for submitting your manuscript to PLOS Neglected Tropical Diseases. After careful consideration, we feel that it has merit but does not fully meet PLOS Neglected Tropical Diseases's publication criteria as it currently stands. Therefore, we invite you to submit a revised version of the manuscript that addresses the points raised during the review process.

Please submit your revised manuscript within by Apr 02 2026 11:59PM. If you will need more time than this to complete your revisions, please reply to this message or contact the journal office at plosntds@plos.org. When you're ready to submit your revision, log on to https://www.editorialmanager.com/pntd/ and select the 'Submissions Needing Revision' folder to locate your manuscript file.

Please include the following items when submitting your revised manuscript:

* A letter that responds to each point raised by the editor and reviewer(s). You should upload this letter as a separate file labeled 'Response to Reviewers'. This file does not need to include responses to any formatting updates and technical items listed in the 'Journal Requirements' section below.

* A marked-up copy of your manuscript that highlights changes made to the original version. You should upload this as a separate file labeled 'Revised Manuscript with Track Changes'.

* An unmarked version of your revised paper without tracked changes. You should upload this as a separate file labeled 'Manuscript'.

If you would like to make changes to your financial disclosure, competing interests statement, or data availability statement, please make these updates within the submission form at the time of resubmission. Guidelines for resubmitting your figure files are available below the reviewer comments at the end of this letter.

We look forward to receiving your revised manuscript.

Kind regards,

Anil Fastenau, M.D., M.Sc.

Guest Editor

PLOS Neglected Tropical Diseases

Stuart Blacksell

Section Editor

PLOS Neglected Tropical Diseases

Shaden Kamhawi

co-Editor-in-Chief

PLOS Neglected Tropical Diseases

orcid.org/0000-0003-4304-636XX

Paul Brindley

co-Editor-in-Chief

PLOS Neglected Tropical Diseases

orcid.org/0000-0003-1765-0002

**Journal Requirements:**

If the reviewer comments include a recommendation to cite specific previously published works, please review and evaluate these publications to determine whether they are relevant and should be cited. There is no requirement to cite these works unless the editor has indicated otherwise.

1) Please provide an Author Summary. This should appear in your manuscript between the Abstract (if applicable) and the Introduction, and should be 150-200 words long. The aim should be to make your findings accessible to a wide audience that includes both scientists and non-scientists. Sample summaries can be found on our website under Submission Guidelines:

https://journals.plos.org/plosntds/s/submission-guidelines#loc-parts-of-a-submission

2) Please upload all main figures as separate Figure files in .tif or .eps format. For more information about how to convert and format your figure files please see our guidelines:

https://journals.plos.org/plosntds/s/figures

3) Some material included in your submission may be copyrighted. According to PLOSu2019s copyright policy, authors who use figures or other material (e.g., graphics, clipart, maps) from another author or copyright holder must demonstrate or obtain permission to publish this material under the Creative Commons Attribution 4.0 International (CC BY 4.0) License used by PLOS journals. Please closely review the details of PLOSu2019s copyright requirements here: PLOS Licenses and Copyright. If you need to request permissions from a copyright holder, you may use PLOS's Copyright Content Permission form.

Please respond directly to this email and provide any known details concerning your material's license terms and permissions required for reuse, even if you have not yet obtained copyright permissions or are unsure of your material's copyright compatibility. Once you have responded and addressed all other outstanding technical requirements, you may resubmit your manuscript within Editorial Manager.

Potential Copyright Issues:

i) Figures 5, and 6. Please (a) provide a direct link to the base layer of the map (i.e., the country or region border shape) and ensure this is also included in the figure legend; and (b) provide a link to the terms of use / license information for the base layer image or shapefile. We cannot publish proprietary or copyrighted maps (e.g. Google Maps, Mapquest) and the terms of use for your map base layer must be compatible with our CC BY 4.0 license.

Note: if you created the map in a software program like R or ArcGIS, please locate and indicate the source of the basemap shapefile onto which data has been plotted.

If your map was obtained from a copyrighted source please amend the figure so that the base map used is from an openly available source. Alternatively, please provide explicit written permission from the copyright holder granting you the right to publish the material under our CC BY 4.0 license.

If you are unsure whether you can use a map or not, please do reach out and we will be able to help you. The following websites are good examples of where you can source open access or public domain maps:

* U.S. Geological Survey (USGS) - All maps are in the public domain. (http://www.usgs.gov)

* PlaniGlobe - All maps are published under a Creative Commons license so please cite u201cPlaniGlobe, http://www.planiglobe.com, CC BY 2.0u201d in the image credit after the caption. (http://www.planiglobe.com/?lang=enl)

* Natural Earth - All maps are public domain. (http://www.naturalearthdata.com/about/terms-of-use/).

4) In the online submission form, you indicated that The datasets generated and/or analyzed during the current study are available from the corresponding author upon reasonable request and with permission from the Uganda Public Health Fellowship Program (UPHFP). . All PLOS journals now require all data underlying the findings described in their manuscript to be freely available to other researchers, either

1. In a public repository

2. Within the manuscript itself

3. Uploaded as supplementary information.

This policy applies to all data except where public deposition would breach compliance with the protocol approved by your research ethics board. If your data cannot be made publicly available for ethical or legal reasons (e.g., public availability would compromise patient privacy), please explain your reasons by return email and your exemption request will be escalated to the editor for approval. Your exemption request will be handled independently and will not hold up the peer review process, but will need to be resolved should your manuscript be accepted for publication. One of the Editorial team will then be in touch if there are any issues.

**Reviewers' Comments:**

Reviewer's Responses to Questions

**Key Review Criteria Required for Acceptance?**

As you describe the new analyses required for acceptance, please consider the following:

**Methods**

-Are the objectives of the study clearly articulated with a clear testable hypothesis stated?

-Is the study design appropriate to address the stated objectives?

-Is the population clearly described and appropriate for the hypothesis being tested?

-Is the sample size sufficient to ensure adequate power to address the hypothesis being tested?

-Were correct statistical analysis used to support conclusions?

-Are there concerns about ethical or regulatory requirements being met?

Reviewer #1: The authors used DHIS2 for their analysis but there is no discussion on data quality, reporting biases that are critical for interpreting the observed trends.

Statistical analysis: The Mann-Kendall test is appropriately chosen for trend analysis, but the results are presented without confidence intervals or measures of variability. Although the authors have reported p-values from their analysis, complementary analysis like Theil-Sen Estimator (Sen's Slope) would be handy in illustrating the trend magnitude (effect size).

Incidence calculation: The use of 2024 population estimates as denominators for all years may introduce inaccuracies. The incidence should be calculated using the estimated mid-year population for each year.

Spatial analysis: The description of GIS methods is minimal. Clustering methods (e.g., Moran’s I) should be applied to statistically identify hotspots rather than relying solely on visual inspection.

A sub-analysis for the West Nile region would be helpful. It would answer the question: Is the incidence leprosy uniformly distributed in the West Nile region? This would enable targeted interventions within the region.

Spatial analysis of the child leprosy cases would add a lot value to this paper, since it would point to where leprosy transmission is on-going.

Reviewer #2: This is a well written section overall with a clear objective. The objective does not conclude a hypothesis and its assessment. Consider explicitly stating a priori expectations or clearly position this as hypothesis-generating.

Could you elaborate on the underlying definitions for "new leprosy cases", describing how PB/MB and disability grading were typically measured and classified, and whether this was assigned at diagnosis.

Although this is a descriptive assessment, could you provide some justifications for the trend-testing approach ou used. It is not clear everywhere that the quarterly data was used since there is mentioning of annual reporting. Correct this, otherwise the trend tests with 5 annual points may be underpowered if no quarterly data was used.

This also affects proper display of applying the Mann-Kendall.

The manuscript states that 2024 census population estimates were used as denominators for incidence/prevalence calculations. Using a single-year denominator for multiple years can bias rates. Consider using annual population estimates, or interpolated annual denominators)

Please add some specific details on the GIS mapping part: This should include the shapefile source, mapping unit, classification approach. Also avoid language using "hotspot" or "clusters" unless it relates to a precise method where this is commonly measured.

There is an internal inconsistency: one section indicates ethics approval was not required because data were anonymized and aggregated, while another states protocols were approved by US CDC IRB and Uganda MoH.

Reviewer #3: The objective is clearly stated.

The study design is sound, but the presentation of the data and the conclusions use obsolete techniques and indicators.

**********

**Results**

-Does the analysis presented match the analysis plan?

-Are the results clearly and completely presented?

-Are the figures (Tables, Images) of sufficient quality for clarity?

Reviewer #1: Table 1 is incomplete; it only shows counts and percentages for sex, type, and G2D but lacks annual breakdowns, or regional data. Annual breakdowns are needed for temporal trends while regional data is needed for spatial trends.

Reviewer #2: Overall, results align broadly with the described descriptive, trend, and spatial mapping approach, but there are presentation inconsistencies that require correction or clarification. See the explanations above on annual or quarterly trend testing. Also look at precise and consistent reporting on incidence vs proportion.

The total in table 1 is not equal the n for less than 15 years and 15 and above years. Please correct and ensure that all subtotals sum to the stated total.

In the age-stratified incidence narrative, there is wording that reads as contradictory (higher incidence in ≥15, then statements suggesting the opposite). Please revise for accuracy and consistency with Figure 2.

The manuscript uses G2D prevalence and regional prevalence (~92%) but appears to be mapping the proportion of new cases with G2D, not population prevalence. Please use correct terminology throughout (proportion among new cases vs prevalence in population).

Key findings are clearly stated. However, readers would benefit from uncertainty measures (CIs) for incidence and proportions, especially when comparing against targets.

Reviewer #3: The authors have the data needed for the analysis (in the national DHIS2 system) but do not use it well.

1. Mapping: it would be much better to use the new WHO tool for geographical analysis - the LEMT, which maps child and adult cases separately, as well as going down to district level, which is much more helpful than the regional level used here. See https://www.who.int/publications/i/item/9789290210474 The LEMT does link directly to DHIS2.

2. Child cases are important as they indicate ongoing transmission, but it is obsolete to focus on the child proportion, which fluctuates too much in relation to changes in the denominator (total cases). It is much better to use the actual number of child cases in situations of decreasing endemicity. Using the LEMT will clearly show the districts (probably not that many) where transmission still occurs, and therefore where intensive case-finding and PEP are required.

3. In general, while disability is important, it is not very useful as an epidemiological indicator. In most cases, the disability rate goes up as incidence goes down and diagnosis is less easy. Figure 6 in this paper is very misleading, as it implies that disability is much worse in the southern regions of Uganda, whereas the denominator (new cases) is so low that the proportion of G2D cases is meaningless. I suggest removing the discussion of G2D. Whoever set a target of 3% for G2D obviously doesn't have much experience of leprosy epidemiology.

A good paper to show how this analysis could be presented is "Iqbal, M., Fastenau, A., Salam, A., Sadia, I. I., Fernandes, I., Murtaza, A., ... & Hasker, E. (2025). Pakistan on the Road to Zero Leprosy, an analysis of routine data for the period 1980–2022: a retrospective cohort study. The Lancet Global Health, 13(8), e1458-e1465."

**********

**Conclusions**

-Are the conclusions supported by the data presented?

-Are the limitations of analysis clearly described?

-Do the authors discuss how these data can be helpful to advance our understanding of the topic under study?

-Is public health relevance addressed?

Reviewer #1: The conclusion is somewhat vague and could be more specific to the study findings.

The limitations of the analysis are not fully described. For instance, there is no discussion/justification for use of 2024 census data to analyze incidence in 2020. Similarly, variations in reporting rates over the years or in different regions that could skew the interpretation of observed trends are not discussed. The spatial analysis assumes homogenous distribution within the region. The limitation of using proportions to analyze trends grade 2 disability especially in regions notifying few cases in not described.

Reviewer #2: The conclusion that leprosy remains endemic and that high child case proportion and G2D burden indicate ongoing transmission and delayed diagnosis is broadly supported by the presented indicators.

Consider adding potential diagnostic misclassification to the limitations, as well as possible reporting changes during or after the CAST.

The paper provides updated national surveillance evidence and highlights geographic heterogeneity and programmatic red flags, such as child cases, G2D. You could strengthen the interpretation by clearly distinguishing true epidemiologic change from surveillance intensity changes.

I want to thank the authors with this highly relevant contribution to public health, which is directly tied to national and WHO targets and program planning. The recommended actions are very relevant.

Reviewer #3: The conclusions are poorly stated, due to the underlying analysis, which is inadequate.

**********

**Editorial and Data Presentation Modifications?**

Use this section for editorial suggestions as well as relatively minor modifications of existing data that would enhance clarity. If the only modifications needed are minor and/or editorial, you may wish to recommend “Minor Revision” or “Accept”.

Reviewer #1: Writing and formatting:

- Several typographical errors and inconsistent spacing exist (e.g., lines 9–10, 25–26).

- Abbreviations should be defined at first use (e.g., CAST in line 65 is defined later in the abbreviations list).

- The references are incomplete in the submitted PDF (many URLs are truncated or non-functional).

- Repetitive content: The background and introduction cover similar ground; consider condensing.

- Targets and indicators: The national targets for child cases (<3%) and G2D (<5%) are clearly stated, but their derivation or alignment with WHO thresholds could be briefly explained.

Reviewer #2: Available upon reasonable request with permission may not meet PLOS expectations for unrestricted access to underlying data.

Reviewer #3: The paper has the right approach, but needs to use updated tools and methods.

**********

**Summary and General Comments**

Use this section to provide overall comments, discuss strengths/weaknesses of the study, novelty, significance, general execution and scholarship. You may also include additional comments for the author, including concerns about dual publication, research ethics, or publication ethics. If requesting major revision, please articulate the new experiments that are needed.

Reviewer #1: The manuscript addresses an important public health issue with national and global relevance, and the analysis is based on a substantial national dataset. However, the current version has significant methodological gaps, incomplete results presentation and structural issues that hinder its scientific rigor and clarity. With careful revision—particularly in the methods, results presentation, and discussion—the study could make a valuable contribution to the leprosy elimination literature. I recommend major revision with the expectation that the authors will:

1. Clarify and expand the methods section, especially regarding data quality, statistical approaches, and spatial analysis.

2. Provide a complete Table 1 with annual and geographic breakdowns.

3. Strengthen the discussion by better integrating spatial and temporal findings and addressing limitations more explicitly.

4. Ensure consistent and accurate use of epidemiological terms (incidence vs. prevalence).

5. Correct formatting, referencing, and language issues throughout.

Reviewer #2: This is a timely national-level assessment using routine surveillance with policy-relevant indicators aligned with national and WHO targets. The inclusion of spatial distribution adds programmatic value, identifying persistent high-burden regions such as West Nile and shifting high G2D areas.

Reviewer #3: This is a very worthwhile project, but the analysis and presentation of results need to be redone.

**********

PLOS authors have the option to publish the peer review history of their article (what does this mean?). If published, this will include your full peer review and any attached files.

If you choose “no”, your identity will remain anonymous but your review may still be made public.

**Do you want your identity to be public for this peer review?**  For information about this choice, including consent withdrawal, please see our Privacy Policy.

Reviewer #1: No

Reviewer #2: No

Reviewer #3: **Yes:** Paul Saunderson

[NOTE: If reviewer comments were submitted as an attachment file, they will be attached to this email and accessible via the submission site. Please log into your account, locate the manuscript record, and check for the action link "View Attachments". If this link does not appear, there are no attachment files.]

**Figure resubmission:**

While revising your submission, we strongly recommend that you use PLOS’s NAAS tool (https://ngplosjournals.pagemajik.ai/artanalysis) to test your figure files. NAAS can convert your figure files to the TIFF file type and meet basic requirements (such as print size, resolution), or provide you with a report on issues that do not meet our requirements and that NAAS cannot fix.

-->After uploading your figures to PLOS’s NAAS tool - https://ngplosjournals.pagemajik.ai/artanalysis, NAAS will process the files provided and display the results in the "Uploaded Files" section of the page as the processing is complete. If the uploaded figures meet our requirements (or NAAS is able to fix the files to meet our requirements), the figure will be marked as "fixed" above. If NAAS is unable to fix the files, a red "failed" label will appear above. When NAAS has confirmed that the figure files meet our requirements, please download the file via the download option, and include these NAAS processed figure files when submitting your revised manuscript.

**Reproducibility:**

To enhance the reproducibility of your results, we recommend that authors of applicable studies deposit laboratory protocols in protocols.io, where a protocol can be assigned its own identifier (DOI) such that it can be cited independently in the future. Additionally, PLOS ONE offers an option to publish peer-reviewed clinical study protocols. Read more information on sharing protocols at https://plos.org/protocols?utm_medium=editorial-email&utm_source=authorletters&utm_campaign=protocols

Attachment Submitted filename: Reviewer Report for Manuscript PNTD.docx

# Author comment: Author response to Decision Letter 1

**DOI:** 10.1371/journal.pntd.0014450.r002

10 Apr 2026

Attachment Submitted filename: Point by Point Response to Reviewers revised 19.3.26.docx

# Peer review: Decision Letter 1

**DOI:** 10.1371/journal.pntd.0014450.r003

**Authors:** Stuart Blacksell, Anil Fastenau, Stuart Blacksell, Anil Fastenau

14 May 2026

PNTD-D-26-00058R1

Temporal  and spatial patterns of Leprosy in Uganda, 2020–2024: A nationwide surveillance analysis

PLOS Neglected Tropical Diseases

Dear Dr. Abbo,

Thank you for submitting your manuscript to PLOS Neglected Tropical Diseases. After careful consideration, we feel that it has merit but does not fully meet PLOS Neglected Tropical Diseases's publication criteria as it currently stands. Therefore, we invite you to submit a revised version of the manuscript that addresses the points raised during the review process.

Please submit your revised manuscript by Jun 13 2026 11:59PM. If you will need more time than this to complete your revisions, please reply to this message or contact the journal office at plosntds@plos.org. When you're ready to submit your revision, log on to https://www.editorialmanager.com/pntd/ and select the 'Submissions Needing Revision' folder to locate your manuscript file.

Please include the following items when submitting your revised manuscript:

* A letter that responds to each point raised by the editor and reviewer(s). You should upload this letter as a separate file labeled 'Response to Reviewers'. This file does not need to include responses to any formatting updates and technical items listed in the 'Journal Requirements' section below.

* A marked-up copy of your manuscript that highlights changes made to the original version. You should upload this as a separate file labeled 'Revised Manuscript with Track Changes'.

* An unmarked version of your revised paper without tracked changes. You should upload this as a separate file labeled 'Manuscript'.

If you would like to make changes to your financial disclosure, competing interests statement, or data availability statement, please make these updates within the submission form at the time of resubmission. Guidelines for resubmitting your figure files are available below the reviewer comments at the end of this letter.

As the corresponding author, your ORCID iD is verified in the submission system and will appear in the published article. PLOS supports the use of ORCID, and we encourage all coauthors to register for an ORCID iD and use it as well. Please encourage your coauthors to verify their ORCID iD within the submission system before final acceptance, as unverified ORCID iDs will not appear in the published article. Only the individual author can complete the verification step; PLOS staff cannot verify ORCID iDs on behalf of authors.

We look forward to receiving your revised manuscript.

Kind regards,

Anil Fastenau, M.D., M.Sc.

Guest Editor

PLOS Neglected Tropical Diseases

Stuart Blacksell

Section Editor

PLOS Neglected Tropical Diseases

Shaden Kamhawi

co-Editor-in-Chief

PLOS Neglected Tropical Diseases

orcid.org/0000-0003-4304-636XX

Paul Brindley

co-Editor-in-Chief

PLOS Neglected Tropical Diseases

orcid.org/0000-0003-1765-0002

**Reviewers' comments:**

Reviewer's Responses to Questions

**Key Review Criteria Required for Acceptance?**

As you describe the new analyses required for acceptance, please consider the following:

**Methods**

-Are the objectives of the study clearly articulated with a clear testable hypothesis stated?

-Is the study design appropriate to address the stated objectives?

-Is the population clearly described and appropriate for the hypothesis being tested?

-Is the sample size sufficient to ensure adequate power to address the hypothesis being tested?

-Were correct statistical analysis used to support conclusions?

-Are there concerns about ethical or regulatory requirements being met?

Reviewer #1: Study Design

The authors state in line 77-78 the objective of the study as to evaluate Uganda's progress towards leprosy elimination. Use of the WHO Leprosy Elimination Monitoring Tool (LEMT) would be more appropriate if they had data for a longer duration. The LEMT would visualize the temporal & spatial trends and clearly show the progress of the regions towards elimination. For instance, which regions have progressed from phase 1 (until interruption of transmission) to phase 2 (Interruption of transmission until elimination of disease). If the authors opt not to use the LEMT, they should at the minimum compare the rates per million with the elimination targets for 2023 or 2025.

Statistical Analysis

In line 121-125, the authors report using annual mid-year population estimates to calculate the incidence rates. They however do not provide a reference [full citation]or actual denominator values making it hard to reproduce the results. The appropriate reference should include the mid-year population estimates together with the disaggregation by sex (male/female), age (<15 and>/= 15 and the population of the regions.

Reviewer #2: (No Response)

Reviewer #3: The Methods are acceptable.

**********

**Results**

-Does the analysis presented match the analysis plan?

-Are the results clearly and completely presented?

-Are the figures (Tables, Images) of sufficient quality for clarity?

Reviewer #1: Does the analysis Presented match the analysis plan?

-Table 1 is incomplete [lacks geographical data] making the spatial analysis presented not transparent.

-Temporal trends on the proportion of child leprosy cases have been presented instead of absolute numbers or incidence of child leprosy cases. The proportion of child leprosy cases can increase even if child cases are stable or declining, if adult cases decline faster [ Or decrease even if child cases are rising, if adult cases rise faster].

-Spatial distribution of the proportion of leprosy cases diagnosed with Grade 2 disability has been presented. However, given the variation in the denominator across the regions, the analysis may be misleading. That is; regions with too few notifications may appear to have a disproportionately higher burden of disability due to leprosy.

Are the figures (Tables, Images) of sufficient quality for clarity?

-The authors present number of annual cases in Table 1. However, in figure 1-3, they presented quarterly incidence rates. Figures 1-3 can thus not be reproduced using data in table 1. Unless the authors are interested in seasonal trends, use of annual cases would increase clarity for the readers.

Reviewer #2: Figure cross-reference confusion. Lines 185–190 describe the spatial distribution of child leprosy cases but repeatedly cite "(Figure 5)" when this content corresponds to Figure 4. This appears to be a residual numbering error from the revision and should be corrected before publication.

Mild overclaim in the Conclusion. Line 291–292 states "an increasing rate of child case detection" as a confirmed finding, but the Mann-Kendall result for the child proportion trend was p = 0.069 — not statistically significant. The language should be softened to "a non-significant upward tendency" to stay consistent with the Results section.

Reviewer #3: The analysis is sound.

**********

**Conclusions**

-Are the conclusions supported by the data presented?

-Are the limitations of analysis clearly described?

-Do the authors discuss how these data can be helpful to advance our understanding of the topic under study?

-Is public health relevance addressed?

Reviewer #1: Discussion:

The authors stated in line 77-78 that the objective of the study is to evaluate the progress towards leprosy elimination. However, the discussion does not clearly show what progress has/has not been made since it is not anchored to elimination targets/ sub-targets. The WHO's "Towards Zero Leprosy" strategy (2021–2030) explicitly targets a 90% reduction in the child case rate per million population by 2030, not a reduction in the proportion of child cases. The authors attempt in lines 241-243 to compare proportions of child leprosy with the 2030 target [in rates per million population, which is confusing. The discussion would be enriched if the authors commented on weather the data [ annual incidence of cases, rate of new cases with G2D per million population and new child cases per million population] compares with the the sub- targets of 2023/ 2025.

Conclusion

In line 291-297, the authors need to clearly indicate if there are any temporal or spatial trends/patterns and what progress has been made towards leprosy elimination (if any).

Reviewer #2: (No Response)

Reviewer #3: The conclusions are reasonable.

**********

**Editorial and Data Presentation Modifications?**

Use this section for editorial suggestions as well as relatively minor modifications of existing data that would enhance clarity. If the only modifications needed are minor and/or editorial, you may wish to recommend “Minor Revision” or “Accept”.

Reviewer #1: (No Response)

Reviewer #2: Numerical inconsistency in the Discussion. The abstract and Table 1 correctly state that 21% of cases presented with G2D, but line 227 in the Discussion states "G2D remained high, averaging 24%." This discrepancy needs a one-line correction.

Reviewer #3: No comment

**********

**Summary and General Comments**

Use this section to provide overall comments, discuss strengths/weaknesses of the study, novelty, significance, general execution and scholarship. You may also include additional comments for the author, including concerns about dual publication, research ethics, or publication ethics. If requesting major revision, please articulate the new experiments that are needed.

Reviewer #1: (No Response)

Reviewer #2: Thank you. The authors handled the major concerns well. Year-specific population denominators are now used correctly. Sen's slope was added alongside Mann-Kendall, which meaningfully improves interpretability. The West Nile sub-analysis (Figure 6) is a genuine addition and not merely cosmetic. The spatial analysis of child cases fills a gap that Reviewer 1 rightly flagged. Case definitions (new case, PB/MB, G2D) are now clearly articulated in the Methods, which was a notable weakness before.

The study addresses a genuine public health need — national leprosy surveillance data from sub-Saharan Africa over a five-year period are rare and valuable. The use of DHIS2 data is appropriate and the limitations are honestly acknowledged. The spatial findings, particularly the persistent West Nile concentration and the child case clustering in Northern and Eastern Uganda, are actionable and well-presented. The journal fit with PLOS NTDs is strong.

After addressing the last minor issues, the manuscript can be accepted for publication.

Reviewer #3: Acceptable.

**********

PLOS authors have the option to publish the peer review history of their article (what does this mean?). If published, this will include your full peer review and any attached files.

If you choose “no”, your identity will remain anonymous but your review may still be made public.

**Do you want your identity to be public for this peer review?**  For information about this choice, including consent withdrawal, please see our Privacy Policy.

Reviewer #1: No

Reviewer #2: No

Reviewer #3: No

[NOTE: If reviewer comments were submitted as an attachment file, they will be attached to this email and accessible via the submission site. Please log into your account, locate the manuscript record, and check for the action link "View Attachments". If this link does not appear, there are no attachment files.]

**Figure resubmission:**

While revising your submission, we strongly recommend that you use PLOS’s NAAS tool (https://ngplosjournals.pagemajik.ai/artanalysis) to test your figure files. NAAS can convert your figure files to the TIFF file type and meet basic requirements (such as print size, resolution), or provide you with a report on issues that do not meet our requirements and that NAAS cannot fix.

After uploading your figures to PLOS’s NAAS tool - https://ngplosjournals.pagemajik.ai/artanalysis, NAAS will process the files provided and display the results in the "Uploaded Files" section of the page as the processing is complete. If the uploaded figures meet our requirements (or NAAS is able to fix the files to meet our requirements), the figure will be marked as "fixed" above. If NAAS is unable to fix the files, a red "failed" label will appear above. When NAAS has confirmed that the figure files meet our requirements, please download the file via the download option, and include these NAAS processed figure files when submitting your revised manuscript.

**Reproducibility:**

To enhance the reproducibility of your results, we recommend that authors of applicable studies deposit laboratory protocols in protocols.io, where a protocol can be assigned its own identifier (DOI) such that it can be cited independently in the future. Additionally, PLOS ONE offers an option to publish peer-reviewed clinical study protocols. Read more information on sharing protocols at https://plos.org/protocols?utm_medium=editorial-email&utm_source=authorletters&utm_campaign=protocols

# Author comment: Author response to Decision Letter 2

**DOI:** 10.1371/journal.pntd.0014450.r004

5 Jun 2026

Attachment Submitted filename: Point by Point Response to Reviewers V4.docx

# Editor report: Decision Letter 2

**DOI:** 10.1371/journal.pntd.0014450.r005

**Authors:** Stuart Blacksell, Anil Fastenau, Stuart Blacksell, Anil Fastenau, Stuart Blacksell, Anil Fastenau

8 Jun 2026

Dear Field Epidemiology fellow Abbo,

We are pleased to inform you that your manuscript 'Temporal  and spatial patterns of Leprosy in Uganda, 2020–2024: A nationwide surveillance analysis' has been provisionally accepted for publication in PLOS Neglected Tropical Diseases.

Before your manuscript can be formally accepted you will need to complete some formatting changes, which you will receive in a follow up email. A member of our team will be in touch with a set of requests.

Please note that your manuscript will not be scheduled for publication until you have made the required changes, so a swift response is appreciated.

IMPORTANT: The editorial review process is now complete. PLOS will only permit corrections to spelling, formatting or significant scientific errors from this point onwards. Requests for major changes, or any which affect the scientific understanding of your work, will cause delays to the publication date of your manuscript.

Should you, your institution's press office or the journal office choose to press release your paper, you will automatically be opted out of early publication. We ask that you notify us now if you or your institution is planning to press release the article. All press must be co-ordinated with PLOS.

Thank you again for supporting Open Access publishing; we are looking forward to publishing your work in PLOS Neglected Tropical Diseases.

Best regards,

Anil Fastenau, M.D., M.Sc.

Guest Editor

PLOS Neglected Tropical Diseases

Stuart Blacksell

Section Editor

PLOS Neglected Tropical Diseases

Shaden Kamhawi

co-Editor-in-Chief

PLOS Neglected Tropical Diseases

orcid.org/0000-0003-4304-636XX

Paul Brindley

co-Editor-in-Chief

PLOS Neglected Tropical Diseases

orcid.org/0000-0003-1765-0002

***********************************************************

p.p1 {margin: 0.0px 0.0px 0.0px 0.0px; line-height: 16.0px; font: 14.0px Arial; color: #323333; -webkit-text-stroke: #323333}span.s1 {font-kerning: none

# Editor report: Acceptance letter

**DOI:** 10.1371/journal.pntd.0014450.r006

**Authors:** Stuart Blacksell, Anil Fastenau, Stuart Blacksell, Anil Fastenau, Stuart Blacksell, Anil Fastenau

Dear Field Epidemiology fellow Abbo,

We are delighted to inform you that your manuscript, "Temporal  and spatial patterns of Leprosy in Uganda, 2020–2024: A nationwide surveillance analysis," has been formally accepted for publication in PLOS Neglected Tropical Diseases.

We have now passed your article onto the PLOS Production Department who will complete the rest of the publication process. All authors will receive a confirmation email upon publication.

The corresponding author will soon be receiving a typeset proof for review, to ensure errors have not been introduced during production. Please review the PDF proof of your manuscript carefully, as this is the last chance to correct any scientific or type-setting errors. Please note that major changes, or those which affect the scientific understanding of the work, will likely cause delays to the publication date of your manuscript. Note: Proofs for Front Matter articles (Editorial, Viewpoint, Symposium, Review, etc...) are generated on a different schedule and may not be made available as quickly.

Soon after your final files are uploaded, the early version of your manuscript will be published online unless you opted out of this process. The date of the early version will be your article's publication date. The final article will be published to the same URL, and all versions of the paper will be accessible to readers.

For Research Articles, you will receive an invoice from PLOS for your publication fee after your manuscript has reached the completed accept phase. If you receive an email requesting payment before acceptance or for any other service, this may be a phishing scheme. Learn how to identify phishing emails and protect your accounts at https://explore.plos.org/phishing.

Thank you again for supporting open-access publishing; we are looking forward to publishing your work in PLOS Neglected Tropical Diseases.

Best regards,

Shaden Kamhawi

co-Editor-in-Chief

PLOS Neglected Tropical Diseases

Paul Brindley

co-Editor-in-Chief

PLOS Neglected Tropical Diseases
